# Editorial: Understanding vulnerability to major depressive disorder

**DOI:** 10.3389/fpsyt.2025.1660181

**Published:** 2025-08-05

**Authors:** Mehmet Cagdas Eker, Ali Saffet Gonul, Thomas Frodl

**Affiliations:** ^1^ Department of Psychiatry & Standardization of Computational Anatomy Techniques for Cognitive and Behavioral Sciences (SoCAT) Lab, Ege University School Medicine, Ege University, Bornova, Türkiye; ^2^ Klinik für Psychiatrie, Psychotherapie und Psychosomatik, Uniklinik Rheinisch-Westfälische Technische Hochschule (RWTH) Aachen, Universitatsklinikum Aachen, Aachen, Germany

**Keywords:** major depressive disorder, vulnerability, vitamin D, hypothyroidism, cognitive functions, exercise, caregiver, general medical condition

Major depressive disorder (MDD) is a complex and multifactorial psychiatric condition that remains a leading cause of disability worldwide. Despite its high prevalence, our understanding of the mechanisms that underlie individual vulnerability to MDD remains incomplete.

This Research Topic, *Understanding Vulnerability to Major Depressive Disorder*, brings together a diverse collection of original research and reviews that deepen our insights into the biological, psychological, social, and behavioral factors contributing to the onset and course of MDD. These articles span multiple methodologies—from animal models to human clinical and observational studies—highlighting the multidimensional nature of vulnerability and offering valuable avenues for both prevention and intervention.

## Biological and neuroendocrine risk factors

Several contributions explore biological mechanisms that may predispose individuals to depression. Yang et al. investigated the preventive and therapeutic effects of vitamin D in a mouse model of adolescent depression, revealing that vitamin D administration mitigated depression-like behaviors despite no significant change in BDNF expression—suggesting that alternative neurobiological pathways may be involved.

Similarly, Osnaya-Brizuela et al. offered a focused review of acquired hypothyroidism, identifying its role in increasing the risk of psychiatric conditions, including depression and anxiety, potentially via dysregulation of the HPA axis, serotonergic function, neuroinflammation, and impaired neurogenesis. Since hypothyroidism is one of the identifiable and potentially treatable biological contributors to depression, consistent monitoring of at-risk individuals and patients with depression may help reduce illness exacerbation and facilitate effective treatment.

The peripartum period has unique biological features that predispose women to depression, in addition to the profound psychosocial transitions it entails. To enhance the mother-infant relationship in depressed mothers, researchers have explored fast-acting and effective treatment options. Raja et al. conducted a comprehensive meta-analysis evaluating zuranolone, one of the approved treatments for postpartum depression. Their study provides compelling evidence that zuranolone is a safe and effective antidepressant with rapid therapeutic benefits.

## Cognitive and behavioral vulnerabilities

A significant portion of this Research Topic focuses on maladaptive cognitive processing and emotional regulation as key elements of vulnerability. Monéger et al. employed an eye-tracking paradigm to investigate depressive self-focus bias following failure. They found that only a subgroup of severely depressed individuals—specifically those with high levels of guilt and self-blame—exhibited this attentional bias, challenging the assumption that self-focus is a universal feature of depression.

Human cognition relies on emotional input for decision-making, as proposed by Damasio’s somatic marker hypothesis (1). In line with this, Tian et al. examined the relationship between alexithymia and neurocognitive functioning—specifically immediate memory—in first-episode, drug-naïve individuals with MDD. They found a negative correlation between immediate memory performance and alexithymia, suggesting that impairments in emotional processing may be linked to early neurocognitive deficits in depression.

Fortuna and Golonka developed the Depersonalization Mechanism Scale (DMS) and demonstrated that depersonalization functions both as a predictor and consequence of depressive symptoms. It was highly associated with maladaptive emotion regulation strategies such as rumination, self-blame, and withdrawal. Their findings suggest that depersonalization may operate as a trait-like, risk-enhancing coping mechanism in emotionally overwhelming contexts—one that also exhibits sex differences. The study highlights the value of incorporating depersonalization assessment in depression screening protocols.

Adding further insight into cognitive-affective vulnerabilities in youth, Zhao examined the mechanisms underlying suicidal ideation among adolescents with depressive symptoms. Their study identified rumination and school adaptation as critical mediators between depressive symptoms and suicidal ideation, with psychological distress acting as a key driver. These findings underscore the importance of targeting emotion regulation and social functioning in school environments when designing early suicide prevention strategies for adolescents.

## Psychosocial and environmental contexts

Preventive strategies and resilience-building are essential in reducing the burden of depression, just as they are in other health domains. Wang et al. present a meta-analysis on the effects of physical exercise on negative emotions, offering robust evidence of its preventive and therapeutic potential for depression.

General medical conditions exert both biological and psychosocial burdens, often increasing the risk of depression. Kubanek et al. provided evidence supporting the need for routine depression screening among patients receiving hemodialysis. Although it remains unclear whether the vulnerability stems more from the biological impact of chronic kidney disease and its treatment or the life-limiting psychosocial consequences, their findings underscore the importance of integrating mental health monitoring into chronic care settings.

Along the same lines, Ai et al. utilized machine learning algorithms in a large longitudinal study to develop a predictive model for depression among middle-aged and elderly individuals with hypertension. Their results revealed that psychosocial and functional variables—such as chronic pain, sleep problems, and social isolation—were as predictive as traditional medical factors. This work highlights the potential of AI-assisted risk models in public health surveillance and clinical screening.

## Cultural perspectives

As mental health systems shift toward community-based care, informal caregivers increasingly bear the responsibility for long-term support. However, many countries lack sufficient psychosocial and financial resources for caregivers. Munie et al. shed light on the psychological burden experienced by caregivers of individuals with severe mental illness in Northwest Ethiopia, documenting high rates of depression, burnout, and social isolation. Their mixed-methods study identified poverty, perceived stigma, patient nonadherence, and lack of social support as key predictors of caregiver depression.

While this Research Topic does not directly address intergenerational or cross-national dynamics, the inclusion of research from diverse geographic and sociocultural contexts—including studies from Ethiopia, China, and Eastern Europe—underscores the global relevance of depressive vulnerability and the importance of culturally attuned mental health strategies.

## Concluding remarks

Taken together, the articles in this Research Topic offer a multifaceted understanding of vulnerability to major depressive disorder, encompassing biological, cognitive, emotional, environmental, and sociocultural domains. The diversity of these studies reflects the heterogeneity of depression itself—an illness that seldom arises from a single cause and often persists due to complex, interacting vulnerabilities.

What emerges is a compelling case for integrative models of risk and resilience. Whether examining the neuroendocrine implications of hypothyroidism, attentional biases linked to guilt, or the psychological toll of caregiving in under-resourced contexts, each study contributes a crucial piece to the broader puzzle of depression. Importantly, many of these contributions go beyond risk identification to propose actionable tools—such as machine learning algorithms and psychometric instruments—that could support early detection and personalized prevention.

This Research Topic also underscores the need to contextualize vulnerability—not only through biological frameworks but also within family systems, social inequalities, and cultural landscapes. Depression is not merely a disruption of neurochemistry; it is a condition fundamentally shaped by how individuals relate to themselves, others, and the world. Accordingly, addressing vulnerability requires a coordinated effort spanning clinical care, research, public health, and policy.

By advancing our understanding of who is vulnerable to depression and why, the research presented here lends support to more personalized, equitable, and preventative mental health strategies. As editors, we hope that the insights offered in this Research Topic will encourage further interdisciplinary collaboration and contribute to a future in which depression is not only more effectively treated—but also more effectively anticipated and, where possible, prevented.
